# Sexual compulsivity and its relationship with condomless sex among unmarried female migrant workers in Shanghai, China: a cross-sectional study

**DOI:** 10.1186/s12905-018-0670-5

**Published:** 2018-11-09

**Authors:** Mengyun Luo, Liping Zhu, Yuanyuan Dong, Zezhou Wang, Qiuming Shen, Dandan Mo, Li Du, Zhiruo Zhang, Yong Cai

**Affiliations:** 1 School of Public Health, Shanghai Jiao Tong University, No.227, South Chongqing Road, Shanghai, 200025 People’s Republic of China; 2 Shanghai Center for Women and Children’s Health, Shanghai, 200062 People’s Republic of China

**Keywords:** Unmarried female migrant workers, Sexual compulsivity, Condomless sex, China

## Abstract

**Background:**

Individuals with high sexual compulsivity are preoccupied with their sexual desire to such an extent that it interferes with their normal daily life and can inhibit self-control. Previous studies have found a close association between sexual compulsivity and condomless sex among different populations; however, no studies have investigated this among unmarried female migrant workers in China. This study aimed to validate the Sexual Compulsivity Scale (SCS) for appropriate use and examine the association between sexual compulsivity and condomless sex in this target population.

**Methods:**

In 2015, we recruited 1325 unmarried female migrant workers in Shanghai, China. Information about sociodemographics, sexual compulsivity, and condomless sex were collected using a structured questionnaire. Exploratory factor analysis and reliability analysis were performed to validate the Chinese version of the SCS. Multiple logistic regression analyses were used to examine the association between sexual compulsivity and condomless sex.

**Results:**

The prevalence of condomless sex in the previous 6 months was 66.8% among all participants. The SCS was proven internally consistent for the overall scale (Cronbach’s α = 0.89), and two factors, Social Disruptiveness (Cronbach’s α = 0.87) and Perceived Self-Control (Cronbach’s α = 0.84), were extracted. With mean total score of 18.25 (standard deviation = 4.94) after adjusting for significant sociodemographic factors, the SCS total scores (adjusted odds ratio [AOR] = 1.04, 95% confidence interval [CI] = 1.02–1.07) and two subscale scores (AOR = 1.07, 95% CI = 1.02–1.13; AOR = 1.06, 95% CI = 1.02–1.10) were all related to inconsistent condom use with partners in the previous 6 months.

**Conclusions:**

The Chinese version of the SCS was found to be well adaptable for use among unmarried Chinese female migrant workers and a potential predictor for condomless sex. In addition to safe sex interventions, counselling on managing sexually compulsivity should also be provided in this population.

## Background

Since 1978, socioeconomic transformation in the wake of China’s opening-up policy has accelerated rural-to-urban migration in the country. As of 2015, there were approximately 247 million internal migrants in China [1]. Nearly half of these migrants were female [2], mostly young, unmarried workers aged around 20 to 24 years old [3].

The immense scale and high mobility of these migrants have raised many public health concerns, including high prevalence of sexually transmitted infections (STIs). Official data for 2015 showed that there were 22,162 new cases of STIs (including HIV/AIDS, hepatitis B virus, gonorrhea, and syphilis) in Shanghai, with migrants accounted for 26.77% of all STI cases and 36.97% of HIV/AIDS cases [4]. There is increasing concern that this large-scale migration might be contributing to the diffusion of STIs by acting as a bridge between low-risk and high-risk individuals in the general population [5–7].

Migrant workers in cities confront marginalized living conditions and inequitable access to medical insurance and health services [8]. Compared with original residents, internal migrants are at an unfair disadvantage in receiving sexual and reproductive health services. This is determined not only by the nature of service provision but also by the overarching social and political environments. On the social side, the vulnerable migrant population is isolated from comprehensive family planning services, and their reproductive health needs are mostly neglected. In the political arena, current national policy mainly targets married couples whereas counseling and services for unmarried women are limited [9].

As a result, many female migrants have insufficient knowledge about how to protect themselves from unwanted pregnancy. The primary indicator of unwanted pregnancy is having sex without use of a condom (condomless sex). Condomless sex and failure to use contraceptive methods reportedly leads to over 70% of unwanted pregnancies in China [10]. About 13 million abortions are induced annually in China, with 49.7% of these among young unmarried females [9–12]. According to a survey conducted in Guangdong Province, nearly one-third of female migrants have undergone induced abortions, 30% of which were illegal abortions at home or in private practice. Illegal abortions can cause particularly severe health consequences, including uterine perforation, intrauterine adhesions, and even death [13, 14].

Several studies have broadly assessed levels of knowledge, attitudes, and behaviors related to consistent condom use, as well as related psychosocial factors such as anxiety, depression, sexual addiction, and loneliness [15–19]. Kalichman [20] noted a relationship between the occurrence of unsafe sexual behavior and a propensity for losing self-control, as in the case of sexual compulsivity. Different from people with sexual addiction and hypersexuality, those with high sexual compulsivity are more likely to experience sexual disinhibition and uncontrolled sexual impulses [21]. Sexual compulsivity has been identified as an inhibitor of safe sexual practices, and might result in profound detrimental health consequences, including transmission of HIV and other sexually transmitted diseases [22–24].

Sexual compulsivity is an intriguing, though unpopular, topic in China as it is seldom discussed among the general public, in what is characteristically a sexually conservative culture. There has only been one study focusing on sexually active Chinese males, in which sexual compulsivity was not found to be associated with condomless sex [25]. However, unmarried females, who are particularly vulnerable to high-risk behavior, may feel greater shame in discussing compulsive sex behavior in China’s largely patriarchal society [26]. Considering the high rate of unwanted pregnancy and induced abortion as a result of condomless sex, there is an urgent need to validate appropriate measures of sexual compulsivity for use among unmarried Chinese female migrant workers, to investigate the impact of sexual compulsivity on condomless sex. We hypothesized that: 1) the Sexual Compulsivity Scale (SCS) is well suited for use among unmarried female migrant workers in China; 2) sexual compulsivity is associated with condomless sex.

## Methods

### Participants

From August to October 2015, we conducted a cross-sectional study among unmarried female migrant workers in three factories in two industrialized areas of Shanghai. All unmarried female workers with any experience of premarital sex behavior were eligible for recruitment. Assuming a prevalence of inconsistent condom use during the previous 6 months of 60% [27, 28], an α of 0.05, and a relative sampling error of 0.1 P, we calculated a required sample size of 900 in case of sampling error and a nonresponse rate of 10%. Of all women who met the criteria, 1325 volunteered to participate. Measures were self-reported in a private room with one investigator and one participant present. After satisfactorily completing the survey, each participant was paid 50 RMB (approximately 7 USD) for her participation.

### Measures

#### Sociodemographic variables

A semi-structured questionnaire was used in this study. Sociodemographic information was collected including age, education level, and monthly income. In addition, participants were asked whether they had smoked tobacco in the previous 30 days (yes or no) and/or had received services related to HIV/AIDS prevention in the previous 6 months (yes or no).

#### Sexual compulsivity variable

The 10-item SCS, designed by Kalichman in 1994 [20], was included in the survey. A four-point Likert-type scale ranging from 1 (strongly disagree) to 4 (strongly agree) was used for rating responses. Total scores ranged from 10 to 40, with a higher score indicating a higher degree of sexual compulsivity. The Chinese scale items were derived from a previous study of the JC School of Public Health and Primary Care, Chinese University of Hong Kong [25].

#### Condomless sex variable

Participants were additionally asked about the frequency of condom use during vaginal intercourse with their partners. Five response choices were given: every time, often, sometimes, seldom, and never. In this study, responses were recoded as a binary variable, with using a condom every time coded as “consistent condom use” and any other responses as “inconsistent condom use”; such response recoding has been widely used in other studies [25, 29]. In the present study, “engaged in condomless sex” was defined as “inconsistent condom use” and was used as the main outcome and the dependent variable.

#### Statistical analysis

Mean and standard deviation (SD) were used for descriptive analysis. Exploratory factor analysis was performed using the principal components method with varimax rotation. Cronbach’s α was used to assess internal reliability. Item analysis was performed by computing the corrected item-to-total correlation coefficients for the overall scale and the subscales. The associations between SCS score and condomless sex were investigated using univariate and adjusted odds ratios (AOR) with their respective 95% confidence intervals (95%CIs). We entered the total score and the two subscale scores separately in three different models. In addition, multiple logistic regression models were fit, adjusting for significant sociodemographic variables related to condomless sex in the unadjusted model. Statistical significance was defined as *p* < 0.05, and IBM SPSS for Windows, Version 22.0 (IBM Corp., Armonk, NY, USA) was used for all statistical analyses.

#### Ethical approval

The study proposal was reviewed and approved by the Chinese National Nature Science Fund Committee. The study was also approved by the Ethics Committee of the School of Public Health, Shanghai Jiao Tong University.

## Results

### Sociodemographic and behavior-related characteristics

The 1325 participants were distributed among three age groups, with 35.0% aged over 23 years old. With respect to education, 69.8% reported receiving formal education for over 9 years. About two-thirds of the sample reported monthly income above 3200 RMB, 7.4% had smoked tobacco in the previous 30 days, and 14.9% had received services related to HIV/AIDS prevention in the previous 6 months. Furthermore, 66.8% of respondents reported engaging in vaginal intercourse without a condom in the previous 6 months (see Table 1).

**Table 1:** Personal characteristics of the study participants (*n* = 1325)

	Number	Percent
Socio-demographic variables		
Age group		
≤21	429	32.4
21–23	432	32.6
> 23	464	35.0
Number of years of formal education		
Primary education (≤9 years)	400	30.2
Secondary education (10–12 years)	841	63.5
Tertiary eduation (>12 years)	84	6.3
Monthly income (Yuan)		
Low (≤3200)	443	33.4
Medium (3201–4800)	828	62.5
High (>4800)	54	4.1
Behavior-related variables		
Smoked tobacco in the past 30 days		
Yes	98	7.4
No	1227	92.6
Having received services related to AIDS prevention in the previous 6 months		
Yes	198	14.9
No	1127	85.1
Engaged in condomless sex in the previous 6 months		
Yes	885	66.8
No	440	33.2

### Survey responses on the SCS

Table 2 shows the frequency distributions of the survey items. Over 20% of participants thought their sexual appetite had gotten in the way of their relationships (Item 1). Meanwhile, 17.2% of female migrant workers found themselves sometimes failing to meet their commitments and responsibilities because of their sexual behaviors (Item 4), and 16.7% felt that their sexual thoughts and feelings were stronger than they were (Item 7).

**Table 2:** Item responses on the Sexual Compulsivity Scale (n = 1325)

Items	N (%)				M	SD
	Strongly agree	Agree	Disagree	Strongly disagree		
1. My sexual appetite has gotten in the way of my relationships	19(1.4)	258(19.5)	687(51.8)	361(27.2)	2.0	0.7
2. My sexual thoughts and behaviors are causing problems in my life	14(1.1)	162(12.2)	772(58.3)	377(28.5)	1.9	0.7
3. My desires to have sex have disrupted my daily life	20(1.5)	151(11.4)	652(49.2)	502(37.9)	1.8	0.7
4. I sometimes fail to meet my commitments and responsibilities because of my sexual behaviors	32(2.4)	196(14.8)	639(48.2)	458(34.6)	1.9	0.8
5. I sometimes get so horny I could lose control	31(2.3)	141(10.6)	674(50.9)	479(36.2)	1.8	0.7
6. I find myself thinking about sex while at work	29(2.2)	115(8.7)	646(48.8)	535(40.4)	1.7	0.7
7. I feel that sexual thoughts and feelings are stronger than I am	34(2.6)	187(14.1)	716(54.0)	388(29.3)	1.9	0.7
8. I have to struggle to control my sexual thoughts and behaviors	27(2.0)	185(14.0)	702(53.0)	411(31.0)	1.9	0.7
9. I think about sex more than I would like to	29(2.2)	94(7.1)	759(57.3)	443(33.4)	1.8	0.7
10. It has been difficult for me to find sex partners who desire having sex as much as I want to	20(1.5)	99(7.5)	748(56.5)	458(34.6)	1.8	0.7

### Psychometric properties of the SCS

Using the factor analysis procedure, we extracted two factors that explained 33.3% and 29.5% of variance (Kaiser–Meyer–Olkin Measure of Sampling Adequacy = 0.91; χ^2^ of Bartlett’s test of sphericity = 5970, *p* < 0.001). The factor loadings are shown in Table 3. The two sexual compulsivity factors were designated Social Disruptiveness (Items 1–4) and Perceived Self-Control (Items 5–10).

**Table 3:** Factor loadings, item–subscale, and item–total correlations of the Sexual Compulsivity Scale (n = 1325)

Items	Factor loadings		Corrected correlation coefficients	
	Factor 1	Factor 2	Item-total	Item-subscale
Factor 1 (Social Disruptiveness)				
1. My sexual appetite has gotten in the way of my relationships	0.82	0.12	0.54	0.65
2. My sexual thoughts and behaviors are causing problems in my life	0.82	0.23	0.64	0.73
3. My desires to have sex have disrupted my daily life	0.81	0.36	0.74	0.79
4. I sometimes fail to meet my commitments and responsibilities because of my sexual behaviors	0.77	0.33	0.67	0.71
Factor 2 (Perceived Self-Control)				
5. I sometimes get so horny I could lose control	0.34	0.63	0.61	0.59
6. I find myself thinking about sex while at work	0.25	0.69	0.60	0.62
7. I feel that sexual thoughts and feelings are stronger than I am	0.11	0.76	0.55	0.61
8. I have to struggle to control my sexual thoughts and behaviors	0.31	0.67	0.62	0.62
9. I think about sex more than I would like to	0.17	0.77	0.61	0.65
10. It has been difficult for me to find sex partners who desire having sex as much as I want to	0.23	0.73	0.61	0.64

Acceptable levels of internal consistency were demonstrated for the SCS. Cronbach’s α was 0.89 for the overall scale, 0.87 for the Social Disruptiveness dimension, and 0.84 for the Perceived Self-Control dimension. The item-to-total, item-to-subscale 1, and item-to-subscale 2 corrected correlation coefficients ranged from 0.54 to 0.74, 0.65 to 0.79, and 0.59 to 0.65, respectively.

### Relationship between SCS and condomless sex

Medium monthly income, having smoked tobacco in the previous 30 days, SCS total score, Social Disruptiveness score, and Perceived Self-Control score were associated with condomless sex in the previous 6 months (*p* < 0.01). After adjusting for the first two significant sociodemographic variables, greater likelihood to engage in condomless sex in the previous 6 months was still related to sexual compulsivity (AOR = 1.04, 95% CI = 1.02–1.07) as well as Social Disruptiveness (AOR = 1.07, 95% CI = 1.02–1.13) and Perceived Self-Control (AOR = 1.06, 95% CI = 1.02–1.10) (see Table 4).

**Table 4:** Factors associated with condomless sex among unmarried female migrant workers in Shanghai, China

	N (row%)/Mean ± Std.	ORu (95%CI)	AOR (95%CI)
Age group			
≤21	287 (66.9)	1	
21–23	301 (69.7)	1.14 (0.85–1.52)	
> 23	297 (64.0)	0.88 (0.67–1.16)	
Number of years of formal education			
Low (≤9)	269 (67.3)	1	
Medium (10–12)	565 (67.2)	1.00 (0.77–1.29)	
High (>12)	51 (60.7)	0.75 (0.46–1.22)	
Monthly income (Yuan)			
Low (≤3200)	274 (61.9)	1	
Medium (3201–4800)	572 (69.1)	1.38 (1.08–1.76) **	
High (>4800)	39 (72.2)	1.60 (0.86–3.00)	
Tobacco use in the past 30 days			
No	804 (65.5)	1	
Yes	81 (82.7)	2.51 (1.47–4.28) **	
Having received services related to AIDS prevention in the past 6 months			
No	743 (65.9)	1	
Yes	142 (71.7)	1.31 (0.94–1.83)	
SCS total	18.25 ± 4.94	1.05 (1.03–1.08) **	1.04 (1.02–1.07) **
Sexual Disruptiveness	7.43 ± 2.40	1.09 (1.03–1.14) **	1.07 (1.02–1.13) **
Perceived Self-Control	10.83 ± 3.13	1.08 (1.04–1.12) **	1.06 (1.02–1.10) **

Dependent variable: condomless sex

AORs were adjusted for monthly income level and tobacco use in the previous 30 days

Abbreviations: *AOR* adjusted odds ratio, *OR* odds ratio, *CI* confidence interval, *SCS* Sexual Compulsivity Scale

** *p* < 0.01

## Discussion

Findings from our study demonstrated that two-thirds of the unmarried female migrant workers surveyed had engaged in vaginal intercourse without a condom in the previous 6 months, which was nearly in accordance with the results of other studies; our results were slightly higher than those for university students and unmarried youth but lower than the results for female sex workers [27, 28, 30, 31]. Owing to the high prevalence of condomless sex and the serious health consequence that might result, there is a clear need to provide this vulnerable population with sexual and reproductive health education.

To our knowledge, ours was the first study specifically targeting sexual compulsivity among unmarried female migrant workers in China, who do not receive sufficient attention with regard to their sexual and reproductive health. We found that the SCS was a highly reliable and valid tool for assessing sexual compulsivity in this target population. Moreover, we found that higher scores for sexual compulsivity as measured by the SCS were related to inconsistent condom use.

The 10-item SCS measure of sexual compulsivity was first developed by Kalichman in 1994 [20]. In the following years, both Kalichman [21] and McBride [32] identified two subscales, which they called Social Disruptiveness (Items 1–4) and Personal Discomfort (Items 5–10). However, the items were grouped differently in studies by Ballester-Arnal [33] and Liao [25]. Ballester-Arnal loaded the 10 items into two factors called Interference of Sexual Behavior (Items 1–4 and 10) and Failure to Control Sexual Impulses (Items 5–9). In the study by Liao in Hong Kong, their modified Chinese version removed Item 4 and consisted of two factors named Functional Consequences (Items 1–3 and 10) and Controllability (Items 5–9). We doubted that the difference might be age and sex dependent, as Ballester’s study mostly included college students aged 18–20 years, and Liao’s only included Chinese males. The present study showed the same results as those of Kalichman and McBride [21, 32]. Adapting the scale items to local Chinese culture, these two dimensions are better labeled Social Disruptiveness and Perceived Self-Control. The present study serves as a reminder that measurement of sexual compulsivity must be culturally adapted and population-based.

Regarding the association between sexual compulsivity and condomless sex, we found that unmarried female migrant workers with high sexual compulsivity were more likely to engage in condomless sex. This was unsurprising, given that the same result has been reported for other target populations, including gays, lesbians, bisexual men and women, HIV-positive populations, and young college students [32–34]. Several theories could explain this phenomenon. As per the presently used scale, a higher score can be seen as: 1) preoccupation with one’s sexual desire and sexual fantasy to a level that interferes with work and normal life; 2) a loss of control over obsessive thoughts and actual sexual behaviors [35, 36]. Individuals with difficulty controlling their impulses may be more likely to engage in condomless sex without considering potential negative outcomes such as STIs or unwanted pregnancy [37]. According to the theory of planned behavior, perceived behavioral control is one of the most important predictors of an individual’s intention and behavior [38]. Evidence has showed that less perceived behavioral control over condom use is correlated with practicing condomless sex [39–45]. Meanwhile, attachment theory has postulated another perspective in that a real person cannot live up to the idealistic, imagined reality that a sexually compulsive/addictive person seeks [46]. Such people therefore tend to use sex as a means of comfort and anxiety reduction, without a need for emotional intimacy, which might also lead to condomless sex [47].

Our study results should be interpreted while considering several limitations. First, we cannot draw causal conclusions owing to the cross-sectional design of the study. Second, the clustered sampling method might result in selection bias; however, this method is commonly used for recruiting in highly aggregated populations, such as that in the present study. Third, given that the sample population was limited to one city, we cannot assert that is representative of all unmarried female migrant workers in China. Fourth, this study was limited in its reliance on self-report data because of the sensitive topic; however, we tried our best to obtain unbiased answers. Fifth, some other interesting elements, such as the type of male sex partners involved, intimate partner violence, and other unwanted sex including with employers or prospective employers, were not measured in this study; these could be included in further study.

Despite these limitations, given the large sample size, this report provides interesting insight on sexual compulsivity and its relationship with condomless sex in this relatively unique population. Further longitudinal research is needed to build on the findings of this study.

## Conclusions

The Chinese version of the Sexual Compulsivity Scale was found to be suitable for use among unmarried Chinese female migrant workers and was evidently a potential predictor for condomless sex. This suggested that substantially more attention should be paid to implementing effective behavioral interventions among highly sexually compulsive female migrant workers in large cities. In addition to education about safe sex, counseling interventions should also be provided, to teach these women how to defuse sexually compulsive behavior and integrate healthy sexual behavior into their daily life.

## Abbreviations

AOR: Adjusted odds ratios
CI: Confidence interval
SCS: Sexual Compulsivity Scale
SD: Standard deviation
STIs: Sexually transmitted infections
